# 25 Years of On-going Endeavors and Experience towards Promoting Iran’s Pharmacy

**Published:** 2013

**Authors:** Hossein Vahidi

The constant endeavors of a group of pharmacists graduated in different areas in Shahid Beheshti University of Medical Sciences has led to the foundation of one of the most prestigious pharmacy schools, not only within Iran, but among Eastern Asian pharmacy Schools. We are proud to state that we have been successfully carrying on a great share of the pharmaceutical advancements in many various relevant fields. This honor would have never been obtained without the on-going supports rendered by the University’s officials, Ministry of Health, and the generous efforts of our treasured faculty members.

